# Prediction Model of Coal Gas Permeability Based on Improved DBO Optimized BP Neural Network

**DOI:** 10.3390/s24092873

**Published:** 2024-04-30

**Authors:** Wei Wang, Xinchao Cui, Yun Qi, Kailong Xue, Ran Liang, Chenhao Bai

**Affiliations:** 1 College of Mechanical Engineering and Automation, Liaoning University of Technology, Jinzhou 121001, China; wangwei@sxdtdx.edu.cn; 2 School of Coal Engineering, Shanxi Datong University, Datong 037000, China; sdtu-faker@sxdtdx.edu.cn (K.X.); 210857002131@sxdtdx.edu.cn (R.L.); 230857002123@sxdtdx.edu.cn (C.B.); 3 China Safety Science Journal Editorial Department, China Occupational Safety and Health Association, Beijing 100011, China

**Keywords:** coal gas, permeability, improved dung beetle optimizer (IDBO), BP neural network (BPNN), prediction model

## Abstract

Accurate measurement of coal gas permeability helps prevent coal gas safety accidents effectively. To predict permeability more accurately, we propose the IDBO-BPNN coal body gas permeability prediction model. This model combines the Improved Dung Beetle algorithm (IDBO) with the BP neural network (BPNN). First, the Sine chaotic mapping, Osprey optimization algorithm, and adaptive T-distribution dynamic selection strategy are integrated to enhance the DBO algorithm and improve its global search capability. Then, IDBO is utilized to optimize the weights and thresholds in BPNN to enhance its prediction accuracy and mitigate the risk of overfitting to some extent. Secondly, based on the influencing factors of gas permeability, effective stress, gas pressure, temperature, and compressive strength, they are chosen as the coupling indicators. The SPSS 27 software is used to analyze the correlation among the indicators using the Pearson correlation coefficient matrix. Additionally, the Kernel Principal Component Analysis (KPCA) is employed to extract the original data. Then, the original data is divided into principal component data for the model input. The prediction results of the IDBO-BPNN model are compared with those of the PSO-BPNN, PSO-LSSVM, PSO-SVM, MPA-BPNN, WOA-SVM, BES-SVM, and DPO-BPNN models. This comparison assesses the capability of KPCA to enhance the accuracy of model predictions and the performance of the IDBO-BPNN model. Finally, the IDBO-BPNN model is tested using data from a coal mine in Shanxi. The results indicate that the predicted outcome closely aligns with the actual value, confirming the reliability and stability of the model. Therefore, the IDBO-BPNN model is better suited for predicting coal gas permeability in academic research writing.

## 1. Introduction

Coal mine gas accidents are a significant concern in the global coal mining safety field, posing a serious threat to both coal production and the safety of workers’ lives [1,2]. Coal gas permeability refers to the ability of gas to transmit through a unit area of coal within a unit of time. It is one of the key parameters for evaluating the potential release of gas from coal reservoirs [3,4]. However, accurately predicting the gas permeability of coal remains a challenging problem due to the heterogeneity and complex geological structure of coal.

Currently, both domestic and international scholars are primarily focused on studying the factors that influence changes in gas permeability [5,6]. Li Bobo et al. [7] conducted research on coal samples from the Liupanshui mining area in Guizhou. They applied the theory of effective stress to conduct seepage tests on coal and rock to investigate the impact of pore pressure changes on the characteristics of coal and rock infiltration. Gong Weidong et al. [8] utilized a triaxial penetration device to conduct tests and concluded that the gas permeability of coal is closely associated with factors such as effective stress, gas pressure, and compressive strength of coal. In recent years, with the advancement of science and technology, machine learning and deep learning have become widely utilized as emerging methods for prediction [9,10]. For instance, Tang Guoshui et al. [11] employed the enhanced Artificial Bee Colony algorithm (ABC) to optimize the kernel function parameters C and γ of the Support Vector Machine (SVM). They developed a permeability prediction model for coal-containing gas based on ABC-SVM. The findings demonstrate strong generalization ability and provide a new perspective for studying the permeability of coal-bearing gas. Shao Liangshan et al. [12] utilized Particle Swarm Optimization (PSO) to optimize the hyperparameters of the Least Squares Support Vector Machine (LSSVM). They developed a gas permeability prediction model called PSO-LSSVM and compared its predictive performance with that of BP Neural Network (BPNN) and SVM to enhance the accuracy of predictions. Xie Lirong et al. [13] utilized Learning Vector Quantization (LVQ) to classify and identify sample parameters. They then optimized the weights and thresholds of the BPNN using an enhanced PSO method. They developed a coal gas permeability prediction model based on LVQ-CPSO-BPNN, which showed the closest predicted values to the actual ones. Wang Pan et al. [14] utilized the Mean Impact Value method (MIV) to analyze the factors influencing coal seam gas permeability. They then developed a more precise prediction model for coal seam gas permeability using BPNN. This research provides valuable insights for the study of coal mine safety production and related fields. Ma Shengxiang et al. [15] employed factor analysis to reduce the dimensionality of the original data, thereby decreasing the number of input layers in the BPNN structure and simplifying it. This led to an improvement in the accuracy of model predictions. Song Xi et al. [16] utilized the Random Forest (RF) algorithm to construct a model for predicting coal gas permeability. The effectiveness of the model was validated through practical engineering tests, demonstrating its applicability in actual production and its significant role in guiding mine safety production. In summary, previous studies have made some progress in predicting coal gas permeability. However, there are still several shortcomings that need to be addressed. For instance, Support Vector Machine (SVM) is only suitable for small sample sizes and lacks the optimal method for determining values. LSSVM compromises the robustness and sparsity of standard SVM.BPNN is prone to getting stuck in local optimal values and has a slow convergence rate, as well as potential “overfitting” issues under certain conditions [17]. RF has limited capability in processing low-dimensional data and may exhibit randomness [18]. PSO is sensitive to parameter selection. Although it converges quickly, it easily falls into local optima. None of these methods address the issue of machine learning models tending to overfit. It is evident that current methods for predicting coal gas permeability have limitations that prevent them from meeting the requirements for accurate prediction in academic research.

In order to enhance the accuracy of predicting the gas permeability of coal bodies, the author improved the Dung Beetle Optimizer (DBO) algorithm to rectify its shortcomings and prevent the “overfitting” issue of BPNN. The enhanced DBO algorithm, referred to as IDBO, was employed to optimize the weights and thresholds in BPNN, leading to the development of a prediction model for coal gas permeability known as IDBO-BPNN. Subsequently, the performance of this model was compared with that of PSO-BPNN, PSO-SVM, PSO-LSSVM, and SSA-BPNN models to validate its prediction accuracy. Finally, the model was applied to a coal mine in Shanxi Province to investigate its practicality and stability further. These efforts aim to provide theoretical references to ensure safe and efficient production in coal mines and address related issues.

## 2. Basic Method Principles

### 2.1. Influence Factors of Gas Permeability in Coal

The influencing factors of gas permeability in coal bodies are highly complex, encompassing coal rock properties, stress states, temperature, gas pressure, gas content, and geological structure. An increase in effective stress leads to a reduction in the gap between coal bodies, subsequently decreasing gas permeability. Conversely, an increase in gas pressure leads to higher molecular flow speeds and increased gas permeability. Furthermore, higher temperatures lead to faster movement rates of gas molecules and, consequently, higher permeability [19]. The compressive strength plays a crucial role in determining the compactness of particle arrangement within the coal. Greater compressive strength corresponds to smaller particle gaps and lower permeability [20]. These non-linear factors interact with each other to collectively determine changes in gas permeability within coal.

### 2.2. BP Neural Network

**Figure 1:**
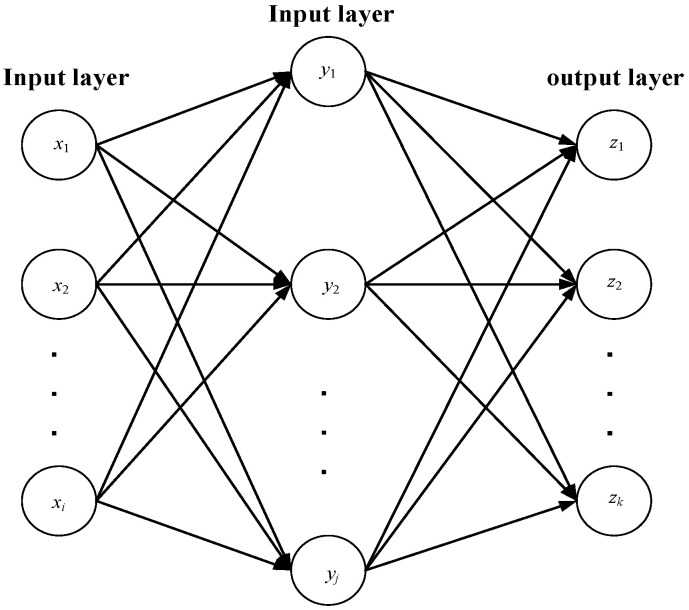
Topology structure of BPNN.

BPNN is a widely used artificial neural network algorithm, typically consisting of three layers of neurons: the input layer, hidden layer, and output layer [21]. The number of nodes in the hidden layer is usually determined by the empirical formula $\sqrt{N_{1}+N_{0}}+L$ where N represents the number of nodes in the hidden layer, *N*_1_ represents the number of nodes in the input layer, and *N*_0_ represents the number of nodes in the output layer [22]. The topology is illustrated in Figure 1.

### 2.3. Improved DBO

#### 2.3.1. DBO

DBO is a novel intelligent optimization algorithm inspired by the rolling, dancing, foraging, stealing, and reproduction behaviors of dung beetles. The algorithm categorizes the dung beetle population into four groups: rolling dung beetle, brooder dung beetle, small dung beetle, and thief dung beetle [23]. Further details can be found in the literature [24].

#### 2.3.2. Improved DBO

Overfitting is a common issue encountered by machine learning models. When the model is too complex, interfered with noise, or when there is limited training data, overfitting is more likely to occur. Therefore, Differential Biogeography Optimization (DBO) is used to optimize the hyperparameters of the Back Propagation Neural Network (BPNN). However, DBO has shortcomings, such as an imbalance in global exploration and local development abilities, which can result in local optimal problems and a weak global exploration ability. To enhance the global search capability of DBO and avoid overfitting BPNN, three strategies are employed to improve DBO. Furthermore, the fitness function is not called multiple times in IDBO. The complexity is consistent with the original DBO.

(1) The population is initialized using the Sine chaotic mapping strategy [25]. The utilization of random generation in the initialization process by intelligent optimization algorithms leads to poor ergodicity, which results in a decrease in the quality of the initial solution [26]. However, utilizing chaotic mapping to generate random numbers significantly enhances the fitness function value and distributes them more evenly. This broader search range helps enhance the accuracy and stability of the algorithm, thereby improving its global search capability. Sine mapping, as a typical representative of chaotic mapping, is simple in form and easy to implement [27]. Its specific formula is as follows:

(1) $$x_{k+1}=\frac{1}{4}\sin(\pi x_{k}),\;a\in (0,4]$$

where *x_k_* is the chaos number of the *k*th iteration.

(2) The Osprey optimization algorithm is introduced in this study. The global exploration strategy of the Osprey optimization algorithm addresses the limitations of the DBO in ball-rolling behavior. The DBO solely relies on the worst value and lacks timely communication with other dung beetles, in addition to having numerous parameters. Therefore, the Osprey optimization algorithm employs a global exploration strategy to randomly locate the position of a dung beetle and update its position by rolling. The specific formula for this strategy is as follows:

(2) $$x_{i}^{P1}=x_{i}+r\cdot (SF-I\cdot x_{i})$$

where $x_{i}^{P1}$ is the new position of the *i* dung beetle in the exploration stage; *r* is the random number between [0, 1]; *SF* is the selected dung beetle and *I* is the random number in the set {1.2}.

(3) Adaptive T-distribution dynamic selection strategy. During the foraging stage of dung beetles, T-distribution perturbations are implemented to influence their foraging behavior. The T-division mutation operator, with the iteration number variation formula serving as the degree of freedom parameter of the T-distribution, is utilized to perturb the foraging behavior. This approach not only makes the best use of current position information but also introduces random interference information, which facilitates escaping from local optimal algorithms [28]. As the number of iterations increases, the T-distribution gradually approaches a Gaussian distribution, thereby enhancing the speed of algorithm convergence. Its mathematical characterization is as follows:

(3) $$x_{new}^{j}=x_{best}^{j}+t(C\_iter)\cdot x_{best}^{j}$$

where $x_{new}^{j}$ is the position of the optimal solution in the *j*th dimension after the adaptive T-distribution variation perturbation; $x_{best}^{j}$ is the position of the optimal solution in the *j*th dimension before the variation perturbation; t(*C*_*iter*) is the degree of freedom parameter of the t distribution.

The introduction of the adaptive T-distribution mutation operator can significantly enhance the optimization performance of the algorithm. However, it is indiscriminately used in all individuals in each iteration, which may lead to an increase in calculation time. Meanwhile, it doesn’t take advantage of the benefits of the original algorithm. To address this issue, a dynamic selection probability P is adopted to adjust the use of adaptive T-distribution mutation operators. This ensures that the algorithm demonstrates strong global development ability in the early stage of iteration while maintaining good local exploration ability in the late stage. Additionally, supplementing the algorithm with T-distribution mutation with a small probability further enhances the convergence speed [29]. The calculation formula for dynamic selection probability P is as follows:

(4) $$P=w_{1}-w_{2}\cdot (Max_{iter}-iter)/Max_{iter}$$

where *w*_1_ is the upper limit of dynamic selection probability; *w*_1_ = 0.5; *w*_2_ is the change amplitude of dynamic selection probability; *w*_1_ = 0.1; *Max_iter_* is the maximum number of iterations; *iter* is the current number of iterations.

#### 2.3.3. Algorithm Validity Test

In order to evaluate the optimization performance of IDBO, the CEC2005 test set is utilized for iterative testing in the Matlab R2023a environment. The algorithm is compared with the Whale Optimization Algorithm (WOA), Subtraction Average Based Optimizer (SABO), Grey Wolf Optimizer (GWO), Northern Goshawk Optimization (NGO), Harris Hawk Optimization (HHO), and the original DBO. Each algorithm’s population size and maximum number of iterations are set to 30 and 1000, respectively, with the test being repeated 30 times. The details of the test function information can be found in Table 1.

**Figure 2:**
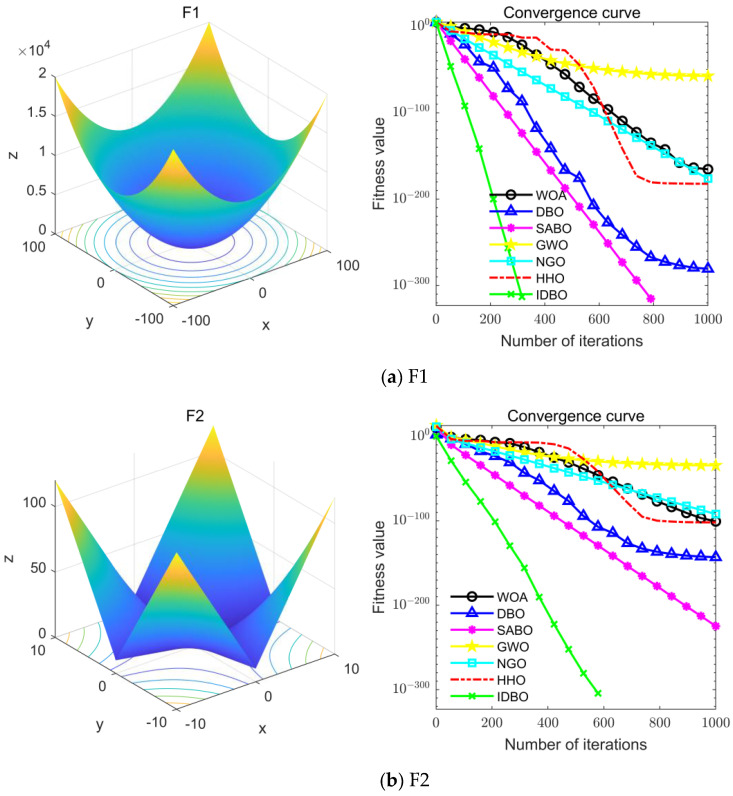

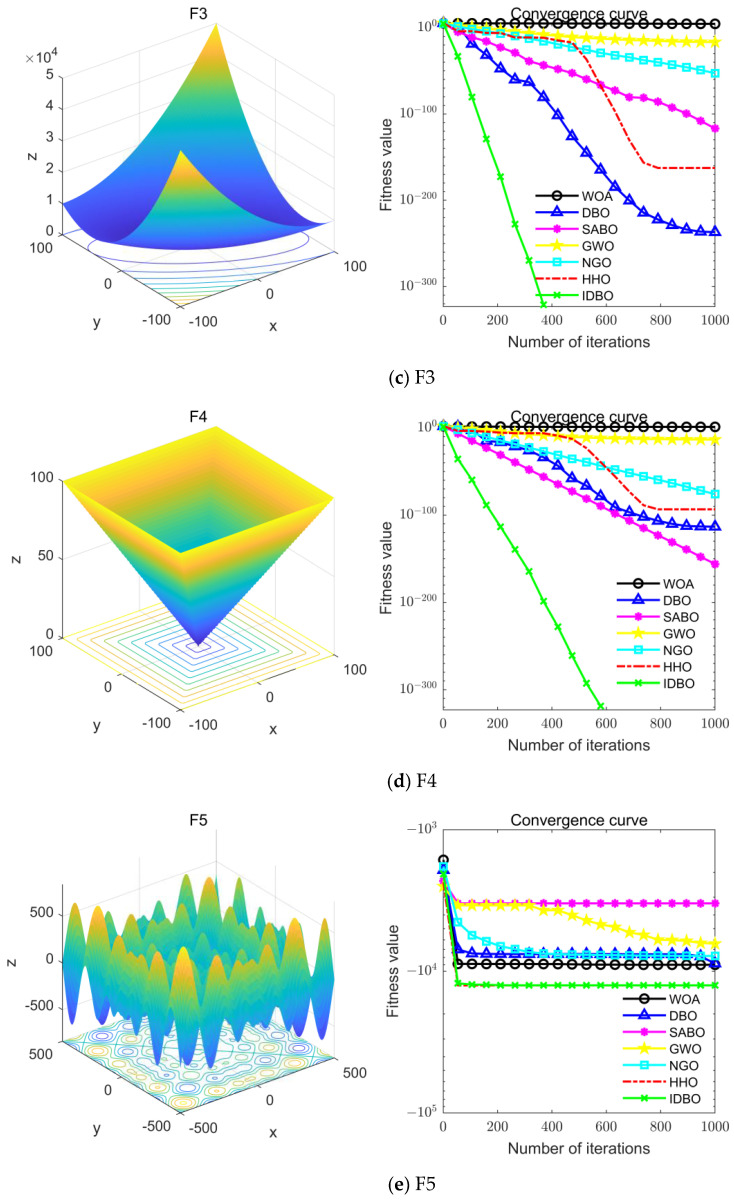

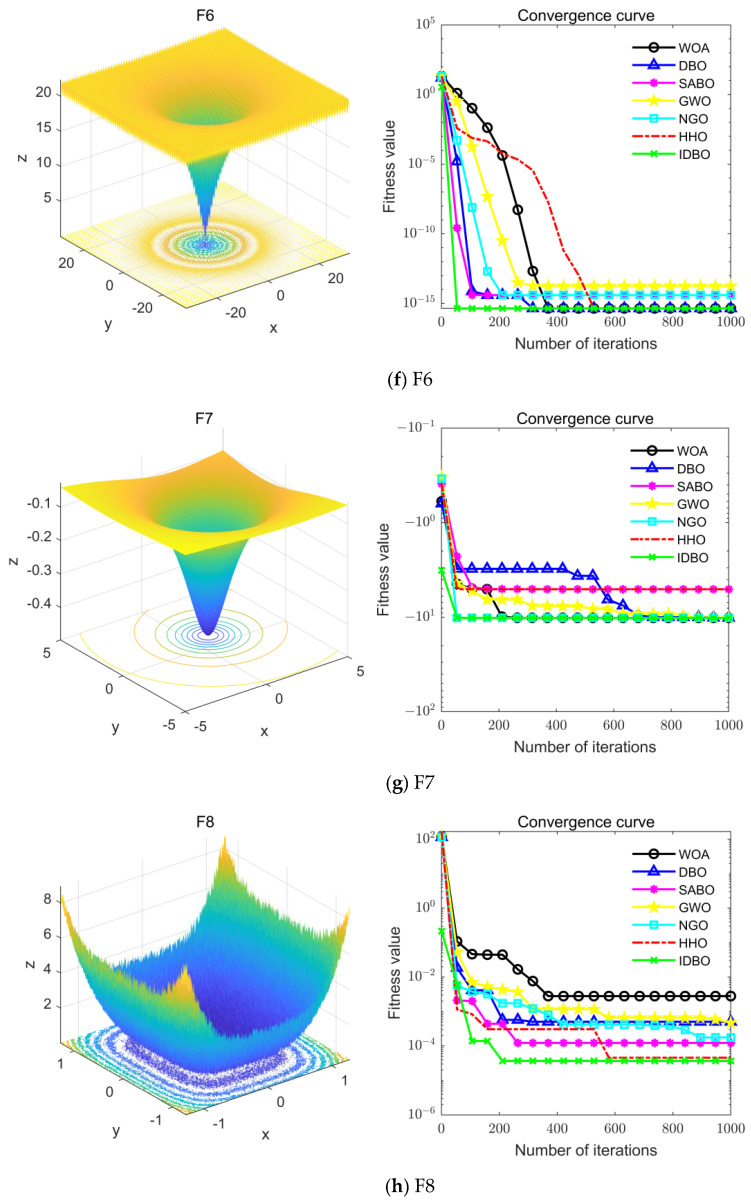
Algorithm convergence curve comparison.

The seven algorithms are tested for comparison and analysis. The test results are shown in Figure 2. The standard test function generates a two-dimensional convergence curve after each algorithm is executed. In this curve, the x-coordinate represents the number of iterations. During each iteration, the algorithm attempts to optimize the function. Therefore, the x-coordinate records the number of these optimization attempts. The goal of CEC test functions is to find the global minimum of the function, so the ordinate usually represents the function value. If the curve slopes downward, it indicates that the algorithm is approaching the optimal solution. If the curve fluctuates greatly, it may suggest that the algorithm is oscillating near the local optimum. According to Figure 2, the slope of the IDBO curve decline is significantly steeper than that of other algorithms in both single-peak benchmark functions and multi-peak, as well as fixed-dimensional multi-peak benchmark functions, which suggests that IDBO exhibits a faster convergence speed. Other algorithms show a relatively gradual decline, indicating that they may be trapped in local optima or experience slow convergence speeds. At the same time, the optimization accuracy of IDBO in test functions F2, F3, F4, F5, F6, F7, and F8 is the best. The fitness value of IDBO in test function F1 is not the best, but it still ranks ahead of several algorithms. The results show that the local development ability of IDBO is significantly improved, which reveals good local development ability compared with the original DBO. In general, IDBO can not only converge quickly but also have the ability to explore and develop balancedly and escape from local optimal solutions.

The seven algorithms are tested by eight different functions with optimal value, standard deviation, average value, median value, and worst value as evaluation indices, which reflect the convergence accuracy and stability of the algorithms, as shown in Table 2. As can be seen from Table 2, IDBO can accurately find the optimal value 0 in various functions, which can adapt to the transformation in global exploration and local exploration. Therefore, compared with other algorithms, IDBO has improved the accuracy of the solution and is more stable in average optimization performance.

Then, the performance of IDBO is further evaluated by the CEC2017 and CEC2021 test sets, as shown in Table 3. It is evident from Table 3 that IDBO has good performance in both the CEC2017 and CEC2021 test sets, showing strong convergence accuracy and speed. In summary, IDBO excellently performs in different test functions. It not only has absolute advantages in convergence speed but also demonstrates good convergence accuracy. At the same time, IDBO achieves a good balance between development and exploration capabilities, which further indicates that IDBO demonstrates outstanding comprehensive performance in many metaheuristic algorithms.

### 2.4. Construction of IDBO-BPNN Model

The metaheuristic optimization algorithm used to optimize machine learning or deep learning models has been demonstrated to significantly improve their prediction accuracy [30]. Therefore, the author utilized Improved Differential Bees Optimization (IDBO) to optimize the weights and thresholds of the Back Propagation Neural Network (BPNN) and established the coal gas permeability prediction model based on IDBO-BPNN. The construction process is illustrated in Figure 2. The specific construction steps are as follows:

(1) Data preprocessing involves handling missing values in the collected data;

(2) Determining whether dimensionality reduction is necessary can be conducted through the Pearson correlation coefficient matrix. If reduction is needed, Kernel Principal Component Analysis (KPCA) can be used to extract principal components from the original data;

(3) Dividing test samples and training samples in a 7:3 ratio and carrying out normalization processing;

(4) Setting the relevant parameters of IDBO and BPNN;

(5) Utilizing the Sine chaotic mapping to initialize the population and calculate the initial fitness value of dung beetles;

(6) Updating the position of each dung beetle and calculating its fitness value to obtain the optimal solution;

(7) Utilizing an adaptive T-distribution dynamic selection strategy to perturb the current optimal solution, acquire a new solution, and assess the need for a position update;

(8) Determining whether termination conditions are met. If not, repeat steps 6–7. If yes, output the optimal parameter;

(9) BPNN acquires optimal weight and threshold parameters for training and simulating predictions.

## 3. Experimental Contrastive Analysis

### 3.1. Data Source and Principal Component Extraction

According to relevant tests and theoretical analysis in the literature [8,31], it is evident that there are numerous factors influencing the gas permeability of coal. The main influencing factors include effective stress, gas pressure, temperature, and coal compressive strength. Therefore, 50 sets of coal gas permeability data under various conditions were selected from the literature [11] as test data for this experiment. Among these groups, data from samples 1 to 40 were used as training samples, while data from 41 to 50 were used as test samples. A portion of the test data is presented in Table 4.

**Figure 3:**
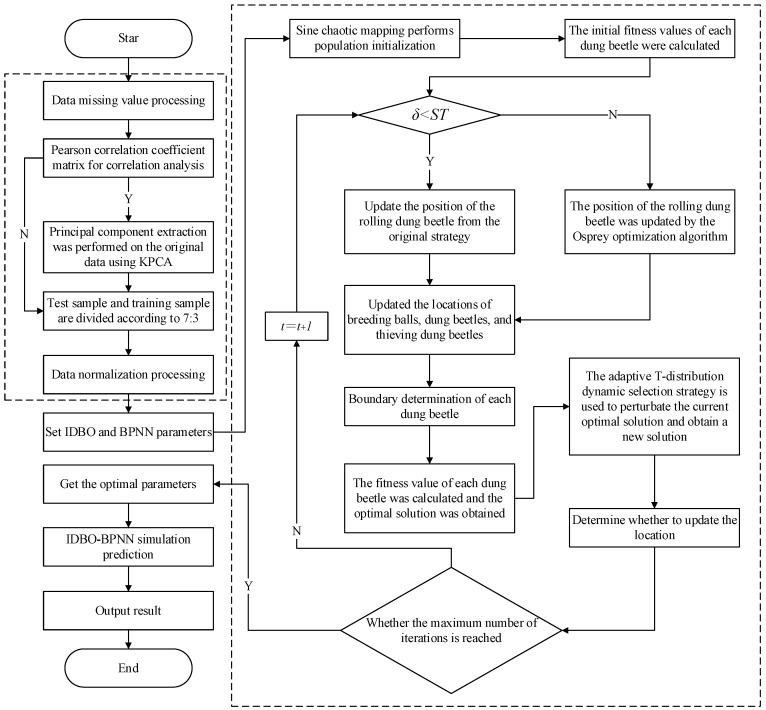
IDBO -BPNN model flow.

**Figure 4:**
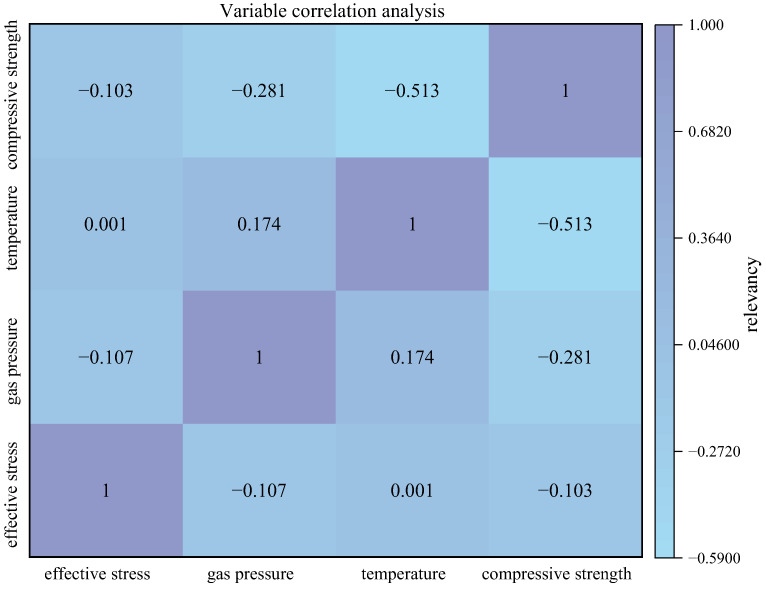
Correlation coefficient matrix.

The correlation analysis chart is a method used to visually represent the distribution of data and the relationship between different factors. In order to accurately capture the impact of different factors, SPSS 27 software was used to perform correlation analysis on the initial data concerning the factors influencing coal gas permeability. This analysis aimed to generate the Pearson correlation coefficient matrix for various indicators, as illustrated in Figure 3. The positive and negative signs in the correlation coefficient indicate the direction of the correlation between variables. A positive correlation coefficient indicates a consistent trend of change between two variables; specifically, when one variable increases, the other variable also increases. A negative correlation coefficient indicates an opposite trend in changes between two variables. This means that when one variable increases, the other variable decreases. According to Figure 4, a negative correlation is observed between effective stress and gas pressure, compressive strength and gas pressure, as well as temperature and compressive strength. Conversely, a positive correlation exists between temperature and effective stress, as well as between temperature and gas pressure. The closer the absolute value of the correlation coefficient is to 1, the stronger the relationship between the variables. A correlation coefficient of 1 indicates a perfect positive correlation, while a correlation coefficient of −1 indicates a perfect negative correlation. A correlation coefficient close to 0 suggests that there is no linear correlation between the two variables. These findings are important for understanding and analyzing relationships between variables in academic research. As shown in Figure 4, the correlation between coal body gas permeability and the influencing factors is not entirely linear; there is a slight correlation between the index factors. For instance, the correlation coefficients between effective stress and gas pressure, temperature, and compressive strength are −0.107, −0.001, and −0.103, respectively. This suggests a limited association among these factors in influencing coal gas permeability. The correlation coefficient between gas pressure and temperature is 0.174. When the correlation value between the two factors is too low (e.g., less than 0.2), it indicates that it may be less helpful for information enrichment. If used directly, it will inevitably affect the result to some extent. Therefore, it is essential to conduct kernel principal component analysis on the original data, which can not only reduce the amount of calculation but also improve the accuracy of model prediction.

Kernel Principal Component Analysis (KPCA) is a nonlinear method for processing data based on a high-dimensional feature space. It involves mapping the data from the original space to a new space and then conducting principal component analysis to successfully achieve dimensionality reduction of linear non-fractional datasets. This technique is widely used in academic research and has proven to be effective in various applications. Due to the nonlinear relationship between the influencing factors of coal gas permeability, Kernel Principal Component Analysis (KPCA) was utilized to reduce the dimensionality of the original data. The selection criteria for this reduction were based on interpreting more than 85% of the cumulative variance. Ultimately, three principal components were extracted and labeled as Y1, Y2, and Y3, respectively. Their respective variance interpretation rates were recorded as 41.74%, 26.83%, and 20.02%. The cumulative interpretive variance is 88.59%, indicating that the three extracted principal components can better reflect the vast majority of information in the original data. Some data after dimensionality reduction are shown in Table 5.

### 3.2. Model Evaluation Index

In order to verify the accuracy and reliability of the constructed prediction model, six indicators are used as the basis to test the prediction accuracy, model advantages and disadvantages, and fitting performance of the prediction model [32]. These indicators include Mean Absolute Error (MAE), Mean Absolute Percentage Error (MAPE), Root Mean Square Error (*RMSE*), R-Square (*R*^2^), Mean Squared Error (*MSE*), and Forecast Bias Ratio (*FBR*). The calculation formulas for these indicators are shown as follows:

(5) $$\mathrm{MAE}=\frac{1}{n}\sum_{i=1}^{n}\left|f_{i}-y_{i}\right|$$

(6) $$\mathrm{MAPE}=\frac{1}{n}\sum_{i=1}^{n}\left|\frac{f_{i}-y_{i}}{y_{i}}\right|\times 100\%$$

(7) $$RMSE=\sqrt{\frac{1}{n}\sum_{i=1}^{n}{\left(f_{i}-y_{i}\right)}^{2}}$$

(8) $$R^{2}=1-\frac{\sum_{i=1}^{n}{\left(y_{i}-f_{i}\right)}^{2}}{\sum_{i=1}^{n}{\left(y_{i}-\bar{y}\right)}^{2}}$$

(9) $$MSE=\frac{1}{n}\sum_{i=1}^{n}{\left(f_{i}-y_{i}\right)}^{2}$$

(10) $$FBR=\frac{y_{i}-f_{i}}{y_{i}}\times 100\%$$

where *n* is the number of samples; *f_i_* is the predicted value; *y_i_* is the true value; $\bar{y}$ is the average of the true values. Among them, the smaller the MAE, MAPE, *RMSE*, and *MSE* values, the closer the *R*^2^ value is to 1, the better, and the closer the *FBR* value is to 0, the better.

### 3.3. Experimental Comparison and Analysis

#### 3.3.1. Multi-Optimization Model Construction

According to the literature [5], the PSO-BPNN model is constructed, and the thresholds and weights of BPNN are optimized using PSO. The PSO-LSSVM model was constructed based on literature [12], and the two parameters *γ* and *σ* in LSSVM were optimized using PSO. Based on reference [33], the PSO-SVM model was constructed, and the penalty parameters and kernel parameters in SVM were optimized using PSO. Additionally, the Marine Predators Algorithm (MPA) optimizing (BPNN) models (MPA-BPNN) was developed based on reference [34]. Furthermore, the WOA-SVM model was developed based on literature [35], while the Bald Eagle Search (BES) optimization SVM model (BES-SVM) was constructed according to reference [36]. These optimization models are compared with IDBO-BPNN and DPO-BPNN models constructed by the author, with parameter settings for each optimization model shown in Table 6.

#### 3.3.2. Comparative Analysis

In the process of fitting and mapping multiple indicators, the significant difference in magnitude between the indicators can directly impact the final result. Therefore, the ‘mapminmax’ function in MATLAB R2023a is used to normalize the original data within a [0, 1] interval. After completing the model simulation and prediction, the mapminmax function is then used to reverse-normalize the data back to its original values. Based on the aforementioned model parameter settings, both the original data and principal component data are used as inputs to obtain permeability prediction results for test samples in each model. The prediction results for the original data are presented in Table 7, while those for the principal component data are shown in Table 8.

By summarizing the aforementioned performance evaluation indicators, the original data evaluation index comparison is shown in Table 9. The comparison of the principal component data evaluation index is shown in Table 10. By comparing the prediction results in Table 7 and Table 8, as well as the performance evaluation indicators in Table 9 and Table 10, principal component extraction of the original data is effectively helpful in concentrating the data, thereby improving the prediction accuracy of the model. Additionally, according to Table 9 and Table 10, the IDBO-BPNN model outperforms other models in various indices. Furthermore, MAE, MAPE, RMSE, *R*^2^, MSE, and FBR of other models in the test samples exhibit significant fluctuations compared to the training samples. This suggests a potential overfitting phenomenon in the test sample stage for these models. As a result, the model’s robustness decreases, and the error of the test sample increases. This further indicates that IDBO enhances the global search capability of the original DBO and improves the prediction accuracy of BPNN. In the case of using the original data, the MAE of the IDBO model in the test stage decreased by 0.0086~0.0271; MAPE decreased by 1.89~3.89%; RMSE decreased by 0.0064~0.0265; and *R*^2^ increased by 0.0188~0.0916 compared with other models. MSE decreased by 0.0008~0.0036; FBR increased by 1.24~4.21%. In the case of using principal component data, the MAE of the IDBO-BPNN model in test samples decreased by 0.0399, 0.0341, 0.0286, 0.0121, 0.021, 0.0188 and 0.0134, respectively, compared with other models. MAPE decreased by 5.61%, 5.55%, 4.19%, 2.01%, 3.14%, 2.5%, 1.95%, and RMSE decreased by 0.0476, 0.0338, 0.0376, 0.0112, 0.023, 0.0185, 0.012, respectively. *R*^2^ was increased by 0.098, 0.0577, 0.0679, 0.0127, 0.033, 0.0244, and 0.0139, respectively, while MSE was decreased by 0.0039, 0.0023, 0.0027, 0.0005, 0.0013, 0.0009, and 0.0005, respectively. FBR decreased by 2.53%, 4.1%, 2.51%, 1.2%, 1.94%, 2.77%, and 1.49%, respectively. Therefore, the IDBO-BPNN model has the smallest error and the best performance.

## 4. Model Case Test

**Figure 5:**
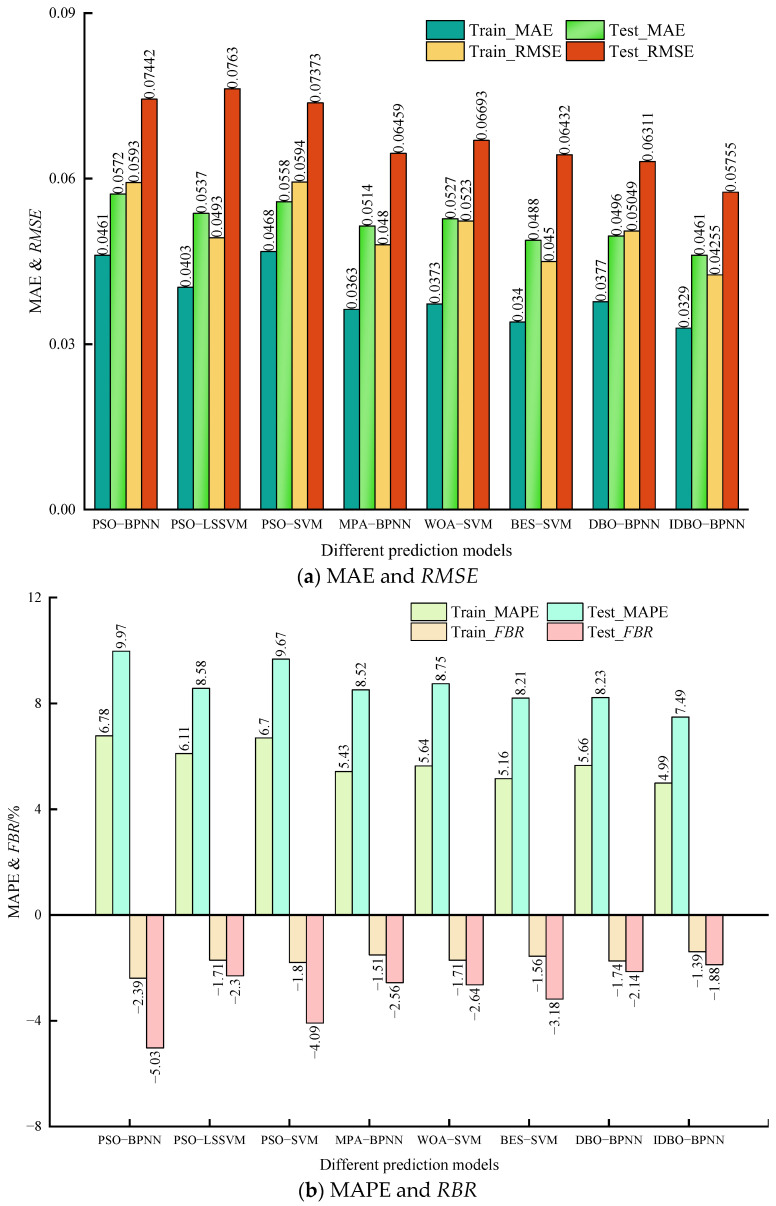

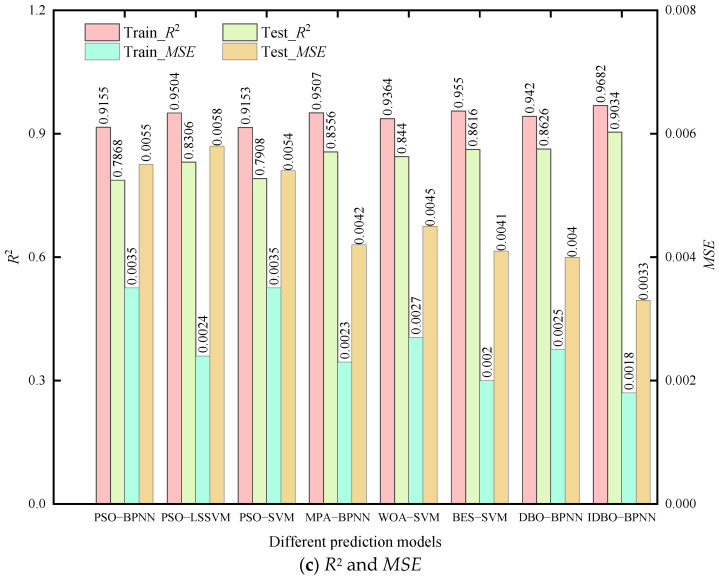
Comparison of evaluation indexes of different models.

In machine learning models, model stability refers to the consistency of performance across various datasets, even when the data is slightly altered or affected by noise. Ensuring the stability of a model is crucial to guarantee its reliability and generalization ability in practical applications. A coal mine in Shanxi Province was selected as the research subject to showcase the reliability and stability of the IDBO-BPNN model. The thickness of No. 2 coal seam in the mine is 0.75~1.93 m, the average thickness is 1.07 m, the coal seam inclination is 3~7°, the absolute emission of gas is 22.23 m^3^/min, the relative emission is 11.74 m^3^/t, it is a high gas mine, not easy to spontaneous combustion coal seam, coal dust is explosive. Therefore, a more accurate prediction of coal gas permeability is essential for preventing gas outburst accidents and ensuring the safe and efficient production of mines. A total of 67 groups of experimental data were selected from the coal mine. Groups 1 to 47 were used as training samples, while groups 48 to 67 were used as test samples. The model parameters remained consistent with the above. First, the Pearson correlation coefficient matrix is used to assess whether the original data needs dimensionality reduction, as shown in Table 11. It is evident from Table 11 that this data requires principal component extraction; therefore, KPCA is still used to process the original data. Finally, three principal components (denoted as Z1, Z2, and Z3) are extracted. Their respective variance interpretation rates are 40.45%, 26.27%, and 19.27%, with a total cumulative variance interpretation rate of 85.99%. Using principal components Z1, Z2, and Z3 as model inputs and permeability as the output variable, the prediction results for each test sample of the model are presented in Table 12. Additionally, the comparison results of performance evaluation indicators for each model are illustrated in Figure 5. As shown in Table 12 and Figure 5, the IDBO-BPNN model developed by the author demonstrates optimal performance in both the training and test samples. In the training sample, the MAE of the IDBO-BPNN model decreased by 0.011~0.139; MAPE decreased by 0.17~1.79%; RMSE decreased by 0.0025~0.0169; *R*^2^ increased by 0.0087~0.0529, compared with other models. MSE decreased by 0.0002~0.0017; FBR decreased by 0.12~1%. In the test sample, the MAE of the IDBO-BPNN model is reduced by 0.0111, 0.0076, 0.0097, 0.0053, 0.0066, 0.0027, and 0.0035, respectively, compared with other models. The MAPE decreased by 2.48%, 1.09%, 2.18%, 1.03%, 1.26%, 0.72%, and 0.74%, while the RMSE decreased by 0.0169, 0.0188, 0.0162, 0.0071, 0.0094, 0.0068, 0.0056, respectively. *R*^2^ was increased by 0.1166, 0.0726, 0.1126, 0.0478, 0.0594, 0.0418, and 0.0408, while MSE was decreased by 0.0022, 0.0025, 0.0021, 0.0009, 0.0012, 0.0008, and 0.0007, respectively. FBR decreased by 3.15%, 0.42%, 2.21%, 0.68%, 0.76%, 1.3%, and 0.26%, respectively. Therefore, the IDBO-BPNN model demonstrates good prediction accuracy and generalization performance.

In conclusion, the IDBO-BPNN model constructed by the author not only demonstrates high prediction accuracy but also exhibits a certain level of reliability and stability. Furthermore, its prediction results are more aligned with reality and can accurately forecast the gas permeability of coal bodies.

## 5. Discussion

(1) In the structural design of the BPNN model, empirical methods are still used to determine the number of hidden layer nodes. However, the verification method for empirical formulas lacks theoretical guidance. Therefore, determining the number of hidden layer nodes in the neural network structure using a scientific and rational method is a future research direction.

(2) The author employs BPNN and SVM as the fundamental models for predicting coal gas permeability. While there are numerous outstanding machine learning and deep learning methods available for developing prediction models, it is essential to conduct further research on combining and comparing these methods in the future.

(3) There are issues such as limited sample data and insufficient verification times. For future studies, it is recommended to select coal samples from different mines and various geological conditions for comparison. This will help to further improve the engineering application capability and universality of the IDBO-BPNN model.

## 6. Conclusions

(1) The integration of Sine chaotic mapping, Osprey optimization algorithm, and adaptive T-distribution dynamic selection strategies into DBO enhances the convergence speed and global search capability of IDBO. Iterative testing was conducted on the CEC2005 test set to validate its performance, comparing it with WOA, SABO, GWO, NGO, HHO, and the original DBO. Further validation was carried out on the CEC2017 and CEC2021 test sets. The results demonstrate that IDBO outperforms other intelligent optimization algorithms in terms of iteration times and accuracy.

(2) A prediction model of gas permeability in a coal body is constructed based on IDBO-BPNN. This model considers the factors influencing gas permeability in a coal body and combines them with IDBO and BPNN. Additionally, a Pearson correlation coefficient matrix analysis was conducted on the original data using SPSS software. The analysis indicated that dimensionality reduction processing was necessary for the original data. Subsequently, principal component extraction was performed on the original data using KPCA, resulting in a cumulative variance of 88.59%.

(3) The original data and principal component data were used as model inputs. The prediction results of the IDBO-BPNN model were compared with those of the PSO-BPNN, PSO-LSSVM, PSO-SVM, MPA-BPNN, WOA-SVM, BES-SVM, and DPO-BPNN models. The results indicate that using the principal component data can effectively improve the model’s prediction accuracy compared to the original data. This suggests that KPCA can effectively help concentrate the data. Secondly, when utilizing principal component data, the MAE of the IDBO-BPNN model in the test samples decreased by 0.0399, 0.0341, 0.0286, 0.0121, 0.021, 0.0188, and 0.0134, respectively, in comparison to other models. The MAPE decreased by 5.61%, 5.55%, 4.19%, 2.01%, 3.14%, 2.5%, and 1.95%. Additionally, the RMSE decreased by 0.0476, 0.0338, 0.0376, 0.0112, 0.023, 0.0185, and 0.012, respectively. *R*^2^ was increased by 0.098, 0.0577, 0.0679, 0.0127, 0.033, 0.0244, and 0.0139, respectively, while MSE was decreased by 0.0039, 0.0023, 0.0027, 0.0005, 0.0013, 0.0009, and 0.0005, respectively. FBR decreased by 2.53%, 4.1%, 2.51%, 1.2%, 1.94%, 2.77%, and 1.49%, respectively. The results indicate that the IDBO-BPNN model demonstrates superior quality, minimal error, and strong fitting performance. Furthermore, it suggests that IDBO significantly enhances global search capability and optimization accuracy compared to the original DBO. As a result, BPNN demonstrates higher prediction accuracy.

(4) To investigate the reliability and stability of the IDBO-BPNN model further, it was applied to a coal mine in Shanxi Province and compared with other prediction models. The results indicate that the IDBO-BPNN model outperforms other models in both training and test samples, demonstrating good prediction accuracy. The result is the closest to the actual value, indicating that the IDBO-BPNN model constructed by the author is more stable and better suited for predicting coal gas permeability. This finding can offer valuable insights for similar mining engineering practices.

## Figures and Tables

**Table 1:** Test function information.

Reference Functions	Dimensionality	Radius
$F1=\sum_{i=1}^{n}x_{i}^{2}$	30	[−100, 100]
$F2=\sum_{i=1}^{n}\left|x_{i}\right|+\prod_{i=1}^{n}\left|x_{i}\right|$	30	[−10, 10]
$F3={\sum_{i=1}^{n}\left(\sum_{j=1}^{i}x_{j}\right)}^{2}$	30	[−100, 100]
$F4=\max_{i}\left\{\left|x_{i}\right|,1\leq i\leq n\right\}$	30	[−100, 100]
$F5=-\sum_{i=1}^{n}\left(x_{i}\sin(\sqrt{\left|x_{i}\right|})\right)$	30	[−500, 500]
$F6=-20\exp\left(-0.2\sqrt{\frac{1}{n}\sum_{i=1}^{n}x_{i}^{2}}\right)$	30	[−32, 32]
$F7=-{\sum_{i=1}^{m}\left[\left(x-a_{i}\right){\left(x-a_{i}\right)}^{T}+c_{i}\right]}^{-1}$	4	[0, 10]
$F8=\sum_{i=1}^{n}ix_{i}^{4}+random\left[0,1\right)$	30	[−1.28, 1.28]

**Table 2:** Comparison of test results.

Functions	Evaluation Criteria	WOA	DBO	SABO	GWO	NGO	HHO	IDBO
F1	Optimal value	2.7 × 10^−101^	2.1 × 10^−191^	1.6 × 10^−241^	4.92 × 10^−36^	1.1 × 10^−108^	7.6 × 10^−132^	0
	Standard deviation	1.03 × 10^−90^	1.5 × 10^−132^	0	1.57 × 10^−33^	8.7 × 10^−106^	5.7 × 10^−109^	0
	Mean value	3.09 × 10^−91^	2.8 × 10^−133^	2.7 × 10^−237^	1.18 × 10^−33^	3.3 × 10^−106^	1.1 × 10^−109^	0
	mid-value	6.86 × 10^−94^	1.8 × 10^−162^	1.5 × 10^−238^	5.89 × 10^−34^	1 × 10^−106^	7.6 × 10^−122^	0
	Worst value	5.47 × 10^−90^	8.3 × 10^−132^	2.5 × 10^−236^	5.73 × 10^−33^	4.8 × 10^−105^	3.1 × 10^−108^	0
F2	Optimal value	5.7 × 10^−106^	5.2 × 10^−206^	2.6 × 10^−242^	1.94 × 10^−36^	3.9 × 10^−109^	8.6 × 10^−133^	0
	Standard deviation	9.38 × 10^−91^	8.5 × 10^−137^	0	2.09 × 10^−34^	9.5 × 10^−107^	6.9 × 10^−113^	0
	Mean value	2.68 × 10^−91^	1.6 × 10^−137^	1.3 × 10^−236^	8.17 × 10^−35^	4.5 × 10^−107^	1.3 × 10^−113^	0
	mid-value	5.7 × 10^−95^	1.7 × 10^−160^	9 × 10^−240^	2.76 × 10^−35^	9.4 × 10^−108^	1.8 × 10^−122^	0
	Worst value	4.81 × 10^−90^	4.7 × 10^−136^	3.4 × 10^−235^	1.09 × 10^−33^	4.2 × 10^−106^	3.8 × 10^−112^	0
F3	Optimal value	3.31 × 10^−68^	1.59 × 10^−94^	4.1 × 10^−137^	2.58 × 10^−21^	6.72 × 10^−57^	2.72 × 10^−67^	0
	Standard deviation	3.19 × 10^−61^	7.59 × 10^−71^	5.3 × 10^−133^	2.75 × 10^−20^	8.67 × 10^−55^	4.38 × 10^−59^	0
	Mean value	7.62 × 10^−62^	1.39 × 10^−71^	1.9 × 10^−133^	2.76 × 10^−20^	5.7 × 10^−55^	8.82 × 10^−60^	1.9 × 10^−300^
	mid-value	2.9 × 10^−65^	5.74 × 10^−83^	2.1 × 10^−134^	1.68 × 10^−20^	2.8 × 10^−55^	9.15 × 10^−64^	0
	Worst value	1.67 × 10^−60^	4.16 × 10^−70^	2.2 × 10^−132^	1.34 × 10^−19^	4.54 × 10^−54^	2.4 × 10^−58^	5.6 × 10^−299^
F4	Optimal value	12,353.02	3.6 × 10^−157^	2.62 × 10^−97^	1.21 × 10^−10^	1.03 × 10^−34^	2.7 × 10^−117^	0
	Standard deviation	12,261.04	2.88 × 10^−65^	1.15 × 10^−51^	7.79 × 10^−7^	2.99 × 10^−27^	6.18 × 10^−72^	0
	Mean value	40,102.65	5.26 × 10^−66^	2.13 × 10^−52^	3.05 × 10^−7^	9.91 × 10^−28^	1.13 × 10^−72^	0
	mid-value	38,697.38	7.6 × 10^−128^	6.67 × 10^−72^	1.14 × 10^−8^	4.27 × 10^−30^	5.5 × 10^−102^	0
	Worst value	64,604.58	1.58 × 10^−64^	6.31 × 10^−51^	3.44 × 10^−6^	1.23 × 10^−26^	3.38 × 10^−71^	0
F5	Optimal value	2.7 × 10^−164^	0	0	9.42 × 10^−67^	1.2 × 10^−210^	1.5 × 10^−262^	0
	Standard deviation	5.3 × 10^−135^	0	0	4.38 × 10^−60^	0	0	0
	Mean value	1.1 × 10^−135^	6.7 × 10^−279^	0	9.29 × 10^−61^	1.7 × 10^−204^	1.9 × 10^−221^	0
	mid-value	1.7 × 10^−145^	0	0	4.92 × 10^−6^3	1.5 × 10^−207^	5.5 × 10^−245^	0
	Worst value	2.9 × 10^−134^	2 × 10^−277^	0	2.4 × 10^−59^	2.6 × 10^−203^	5.8 × 10^−220^	0
F6	Optimal value	2.9 × 10^−160^	5.6 × 10^−216^	0	6.6 × 10^−129^	2.4 × 10^−224^	1.4 × 10^−177^	0
	Standard deviation	3.9 × 10^−130^	9.2 × 10^−137^	0	9.4 × 10^−111^	0	2.7 × 10^−147^	0
	Mean value	8.4 × 10^−131^	1.7 × 10^−137^	2.2 × 10^−302^	1.7 × 10^−111^	4.9 × 10^−217^	7.1 × 10^−148^	0
	mid-value	1.1 × 10^−138^	8.4 × 10^−170^	3 × 10^−308^	7.7 × 10^−122^	2.8 × 10^−220^	4.9 × 10^−157^	0
	Worst value	2.1 × 10^−129^	5.1 × 10^−136^	4.4 × 10^−301^	5.2 × 10^−110^	1.2 × 10^−215^	1.1 × 10^−146^	0
F7	Optimal value	2.15 × 10^−47^	3.27 × 10^−70^	0.099873	0.099873	0.099873	2.98 × 10^−66^	0
	Standard deviation	0.059587	0.042932	1.28 × 10^−07^	0.055086	1.95 × 10^−13^	5.98 × 10^−58^	0
	Mean value	0.129878	0.075022	0.099873	0.179873	0.099873	1.86 × 10^−58^	0
	mid-value	0.099873	0.099873	0.099873	0.199873	0.099873	7.44 × 10^−62^	0
	Worst value	0.299873	0.099873	0.099874	0.299873	0.099873	2.73 × 10^−57^	0
F8	Optimal value	0	0.009716	0.009716	0.009716	0.009716	0	0
	Standard deviation	0.018154	2.79 × 10^−08^	7.25 × 10^−08^	0.013327	5.29 × 10^−14^	0	0
	Mean value	0.022947	0.009716	0.009716	0.034005	0.009716	0	0
	mid-value	0.009716	0.009716	0.009716	0.037224	0.009716	0	0
	Worst value	0.078189	0.009716	0.009716	0.078189	0.009716	0	0

**Table 3:** Optimization curves for different test sets.

Test Set Type	Functions	Convergence Curves	Radius
CEC2017	Shifted and Rotated Rosenbrock’s Function	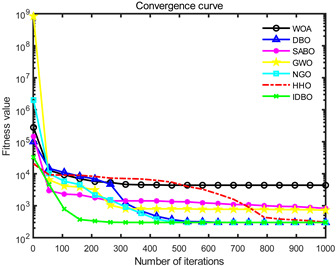	[−100, 100]
	Shifted and Rotated Rastrigin’s Function	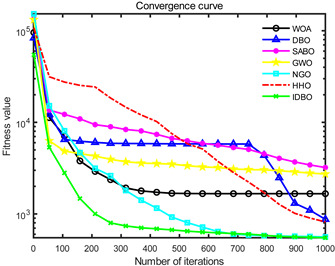	
	Shifted and Rotated Levy Function	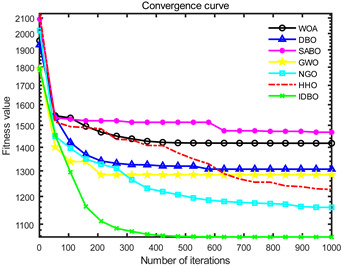	
	Hybrid Function (*N* = 3)	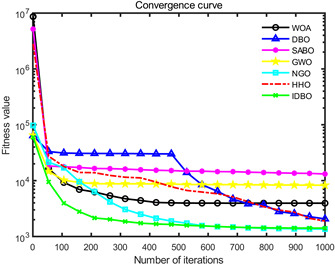	
CEC2021	Shifted and Rotated Bent Cigar Function	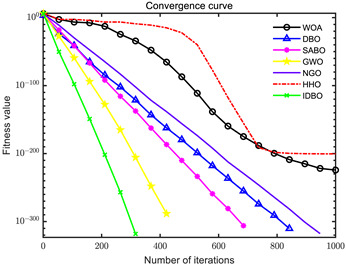	
	Shifted and Rotated Lunacek bi-Rastrigin Function	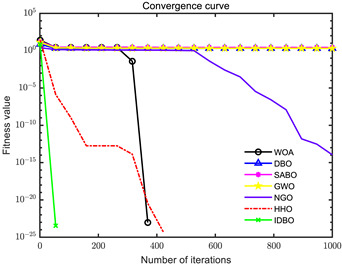	
	Hybrid Function (*N* = 5)	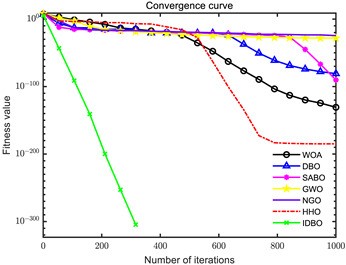	
	Composition Function (*N* = 3)	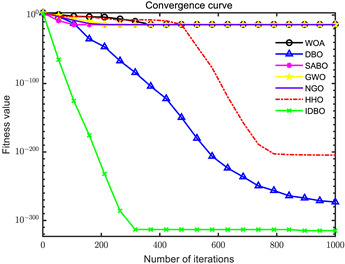	

**Table 4:** Coal gas permeability sample data.

No.	Effective Stress/MPa	Gas Pressure/MPa	Temperature/°C	Compressive Strength/MPa	Permeability/(10^−5^ m^2^)
1	2	1.8	40	10.85	0.881
2	1.51	0.5	55	12.85	1.062
3	4.01	0.5	30	14.13	0.559
…	…	…	…	…	…
24	1.73	1.8	45	14.13	0.805
25	2	1	60	12.62	0.633
26	2.5	1.5	30	12.37	0.677
…	…	…	…	…	…
48	3.78	1	30	12.85	0.491
49	1.73	0.5	30	14.13	1.189
50	2	1	70	11.5	0.632

**Table 5:** KPCA dimension reduction data.

No.	Y1	Y2	Y3	Permeability/(10^−5^ m^2^)
1	0.615	−0.972	1.635	0.881
2	−0.404	−0.453	−0.373	1.062
3	−0.497	2.050	−2.133	0.559
…	…	…	…	…
24	−0.906	−1.783	−0.342	0.805
25	0.173	−0.330	0.496	0.633
26	−0.190	−0.404	−0.053	0.677
…	…	…		…
48	0.092	1.489	−0.806	0.491
49	−1.578	−0.560	−2.232	1.189
50	0.967	−0.088	1.646	0.632

**Table 6:** The parameters of each model are set.

Parameter Name	Specific Setting	Parameter Name	Specific Setting
Population size	30	Maximum iterations	100
BPNN training times	1000	BPNN target error	1 × 10^−6^
BPNN learning rate	0.01	BPNN hidden layer node	12
SVM cross-validate parameters	5	SVM option.gap	0.9
SVM option.cbound	[1, 100]	SVM option.gbound	[1, 100]
PSO learning factor	1.5	PSO inertia weight	0.8
PSO maximum speed limit	1	PSO Maximum speed limit PSO minimum speed limit	−1
MPA FADs	0.2	Probability of WOA contraction enveloping mechanism	[0.1]
WOA spiral position update probability	[0.1]	Variation range of BES spiral trajectory	(0.5, 2)
BES position change parameters	(1.5, 2)	BES spiral trajectory parameters	(0, 5)

**Table 7:** Raw data predicted results.

No.	True Value	Predicted Value							
		PSO-BPNN	PSO-LSVM	PSO-SVM	MPA-BPNN	WOA-SVM	BES-SVM	DBO-BPNN	IDBO-BPNN
40	0.891	0.759	0.863	0.804	0.797	0.820	0.801	0.815	0.803
41	0.516	0.548	0.635	0.582	0.584	0.582	0.579	0.588	0.552
42	0.619	0.525	0.582	0.585	0.609	0.600	0.613	0.608	0.611
43	0.632	0.569	0.612	0.613	0.635	0.629	0.641	0.635	0.642
45	0.564	0.602	0.711	0.676	0.680	0.704	0.691	0.683	0.665
46	0.786	0.724	0.867	0.840	0.811	0.870	0.841	0.844	0.865
47	0.683	0.732	0.705	0.784	0.740	0.689	0.670	0.736	0.688
48	0.491	0.412	0.534	0.518	0.544	0.544	0.538	0.518	0.487
49	1.189	1.044	1.171	1.070	1.163	1.146	1.113	1.127	1.151
50	0.632	0.632	0.727	0.704	0.690	0.725	0.695	0.703	0.686

**Table 8:** Principal component data prediction results.

No.	True Value	Predicted Value							
		PSO-BPNN	PSO-LSSVM	PSO-SVM	PSO-BPNN	WOA-SVM	BES-SVM	PSO-BPNN	IDBO-BPNN
40	0.891	0.746	0.856	0.805	0.850	0.829	0.834	0.830	0.850
41	0.516	0.541	0.472	0.542	0.500	0.505	0.520	0.479	0.518
42	0.619	0.651	0.589	0.607	0.634	0.639	0.558	0.613	0.621
43	0.632	0.685	0.628	0.631	0.637	0.650	0.596	0.620	0.626
45	0.564	0.644	0.498	0.681	0.521	0.514	0.542	0.545	0.558
46	0.786	0.788	0.723	0.864	0.759	0.712	0.750	0.812	0.789
47	0.683	0.759	0.627	0.698	0.676	0.676	0.703	0.653	0.659
48	0.491	0.512	0.553	0.503	0.522	0.518	0.493	0.513	0.511
49	1.189	1.134	1.174	1.165	1.186	1.185	1.153	1.167	1.190
50	0.632	0.655	0.554	0.659	0.587	0.582	0.607	0.622	0.625

**Table 9:** Comparison of raw data evaluation indicators.

Models	Model Performance											
	MAE		MAPE/%		RMSE		*R* ^2^		MSE		FBR/%	
	Train	Test	Train	Test	Train	Test	Train	Test	Train	Test	Train	Test
PSO-BPNN	0.0564	0.0695	7.35	9.65	0.0775	0.0815	0.8568	0.8318	0.0060	0.0066	4.83	5.61
PSO-LSSVM	0.0542	0.0608	8.34	10.00	0.0705	0.0751	0.8817	0.8573	0.0050	0.0056	−5.44	−7.27
PSO-SVM	0.0526	0.0692	7.38	9.95	0.0679	0.0770	0.8903	0.8499	0.0046	0.0059	−2.56	−4.30
MPA-BPNN	0.0457	0.0510	6.65	8.00	0.0569	0.0614	0.9230	0.9046	0.0032	0.0038	−1.49	−5.14
WOA-SVM	0.0462	0.0576	6.75	8.95	0.0582	0.0703	0.9193	0.8748	0.0034	0.0049	−2.64	−5.93
BES-SVM	0.0486	0.0548	7.00	8.19	0.0589	0.0657	0.9173	0.8907	0.0035	0.0043	−1.31	−4.33
DBO-BPNN	0.0447	0.0551	6.54	8.27	0.0570	0.0639	0.9225	0.8966	0.0033	0.0041	−2.60	−5.17
IDBO-BPNN	0.0397	0.0424	5.60	6.11	0.0534	0.0550	0.9319	0.9234	0.0029	0.0030	−0.46	−3.06

**Table 10:** Comparison of evaluation indexes of principal component data.

Models	Model Performance											
	MAE		MAPE/%		RMSE		*R* ^2^		MSE		FBR/%	
	Train	Test	Train	Test	Train	Test	Train	Test	Train	Test	Train	Test
PSO-BPNN	0.0327	0.0511	4.63	7.27	0.0405	0.0644	0.9609	0.8949	0.0016	0.0042	−0.62	−3.10
PSO-LSSVM	0.0167	0.0453	2.56	7.21	0.0422	0.0506	0.9575	0.9352	0.0018	0.0026	1.07	4.67
PSO-SVM	0.0388	0.0398	5.52	5.85	0.0497	0.0544	0.9411	0.9250	0.0025	0.0030	−0.49	−3.08
MPA-BPNN	0.0120	0.0233	1.70	3.67	0.0200	0.0280	0.9905	0.9802	0.0004	0.0008	−0.35	1.77
WOA-SVM	0.0114	0.0322	1.80	4.80	0.0286	0.0398	0.9805	0.9599	0.0008	0.0016	−0.18	2.51
BES-SVM	0.0175	0.0300	2.49	4.16	0.0311	0.0353	0.9770	0.9685	0.0010	0.0012	1.21	3.34
DBO-BPNN	0.0130	0.0246	1.91	3.61	0.0237	0.0288	0.9866	0.9790	0.0006	0.0008	1.02	2.06
IDBO-BPNN	0.0045	0.0112	0.70	1.66	0.0074	0.0168	0.9987	0.9929	0.0001	0.0003	−0.02	0.57

**Table 11:** Pearson correlation coefficient matrix.

	Effective Stress	Gas Pressure	Compressive Strength	Compressive Strength
Effective stress	1	−0.062	0.056	−0.122
Gas pressure	−0.062	1	0.229	−0.230
Compressive strength	0.056	0.229	1	−0.434
Compressive strength	−0.122	−0.230	−0.434	1

**Table 12:** Each model tested the prediction results of the sample.

No.	True Value	Predicted Value							
		PSO-BPNN	PSO-LSSVM	PSO-SVM	MPA-BPNN	WOA-SVM	BES-SVM	DBO-BPNN	IDBO-BPNN
48	0.516	0.527	0.556	0.516	0.564	0.578	0.567	0.570	0.561
49	0.810	0.834	0.762	0.836	0.840	0.828	0.845	0.827	0.839
50	0.516	0.572	0.568	0.552	0.581	0.566	0.576	0.572	0.567
51	0.514	0.564	0.527	0.554	0.557	0.570	0.551	0.562	0.538
52	0.511	0.557	0.522	0.550	0.516	0.520	0.517	0.518	0.533
53	1.056	1.032	0.832	1.034	0.945	0.929	0.931	0.945	0.935
54	0.489	0.545	0.522	0.537	0.516	0.520	0.519	0.518	0.516
55	0.680	0.649	0.718	0.658	0.742	0.762	0.718	0.752	0.718
56	0.845	0.925	0.844	0.927	0.872	0.869	0.871	0.863	0.853
57	0.645	0.572	0.575	0.560	0.608	0.615	0.616	0.602	0.602
58	0.431	0.667	0.616	0.667	0.598	0.602	0.616	0.590	0.560
59	0.580	0.676	0.649	0.677	0.598	0.602	0.616	0.590	0.595
60	0.768	0.671	0.718	0.677	0.758	0.762	0.777	0.769	0.775
61	0.478	0.547	0.532	0.540	0.549	0.538	0.547	0.535	0.557
62	0.745	0.704	0.691	0.695	0.643	0.645	0.669	0.641	0.673
63	0.850	0.802	0.823	0.808	0.781	0.813	0.793	0.796	0.763
64	0.834	0.862	0.801	0.863	0.817	0.799	0.826	0.802	0.792
65	0.654	0.688	0.629	0.683	0.595	0.578	0.606	0.587	0.608
66	0.567	0.544	0.567	0.537	0.515	0.518	0.518	0.517	0.533
67	0.582	0.561	0.628	0.532	0.589	0.575	0.582	0.580	0.585

## Data Availability

All data generated or analyzed during this study are included in this published article and its Supplementary Information Files.

## Supplementary Materials

The following supporting information can be downloaded at: https://www.mdpi.com/article/10.3390/s24092873/s1.
