# Supplementary material for: Prediction Model of Coal Gas Permeability Based on Improved DBO Optimized BP Neural Network
Source: Sensors (Basel). 2024 Apr 30;24(9):2873. doi: 10.3390/s24092873 (PMC11086105; doi:10.3390/s24092873)
Supplement: Supplementary file 1 [file sensors-24-02873-s001.zip › sensors-2936473-supplementary.pdf]

Coal gas permeability sample data

NO.	Effective stress /MPa	Gas pressure /MPa	Temperature /°C	Compressive strength /MPa	Permeability /($10^{-5}m^2$)
1	2	1.8	40	10.85	0.881
2	1.51	0.5	55	12.85	1.062
3	4.01	0.5	30	14.13	0.559
4	2.5	0.4	70	12.62	0.631
5	4.01	0.5	50	14.13	0.46
6	3	1	30	12.85	0.565
7	3.62	1	50	11.62	0.479
8	2	1	30	12.85	0.81
9	3	1	70	11.5	0.451
10	1.23	1.5	40	13.5	0.972
11	1.95	1.2	40	11.62	0.782
12	3.78	0.6	20	12.5	0.617
13	3.78	1	70	11.5	0.402
14	2.5	1.5	50	12.7	0.54
15	1.68	0.7	35	12.85	1.028
16	3	0.5	40	10.62	0.791
17	1.23	1.5	30	12.37	1.023
18	2.64	1.3	65	11.62	0.504
19	3	1.7	60	12.37	0.534
20	2.5	1	30	12.85	0.656
21	2.04	1.6	50	12.85	0.635
22	2.5	1	50	11.62	0.588
23	2	1.5	30	12.37	0.769
24	1.73	1.8	45	14.13	0.805
25	2	1	60	12.62	0.633
26	2.5	1.5	30	12.37	0.677
27	2.5	0.5	30	14.13	0.801
28	3	1.2	40	12.5	0.499
29	3.29	2	60	11.37	0.775
30	3	1.9	55	12.85	0.613
31	2.5	0.5	25	14.15	0.863
32	2	1.6	60	12.37	0.608
33	1.45	1.5	35	14.12	0.955
34	1.75	0.5	45	12.37	1.004
35	2	1	50	11.62	0.724
36	1.83	1.2	55	13.47	0.705
37	3	1	50	11.62	0.561
38	2	0.5	30	14.13	1.042
39	1.48	0.7	30	14.15	1.236
40	1.48	1	30	12.85	1.054

41	1.62	1	50	11.62	0.891
42	2.5	1	70	11.5	0.516
43	3.29	1.5	30	12.37	0.619
44	3	1.5	30	12.37	0.632
45	2.2	1.2	70	11.5	0.564
46	1.45	1	70	11.5	0.786
47	3	0.5	30	14.13	0.683
48	3.78	1	30	12.85	0.491
49	1.73	0.5	30	14.13	1.189
50	2	1	70	11.5	0.632

KPCA dimension reduction data

NO.	Y1	Y2	Y3	Permeability /($10^{-5}m^2$)
1	0.615	-0.972	1.635	0.881
2	-0.404	-0.453	-0.373	1.062
3	-0.497	2.050	-2.133	0.559
4	0.561	0.909	0.337	0.631
5	0.049	2.171	-1.315	0.46
6	-0.278	0.596	-0.840	0.565
7	1.135	1.626	0.819	0.479
8	-0.752	-0.548	-0.883	0.81
9	1.442	1.057	1.689	0.451
10	-1.046	-1.980	-0.446	0.972
11	0.111	-0.548	0.534	0.782
12	-0.098	1.890	-1.376	0.617
13	1.812	1.950	1.723	0.402
14	0.201	-0.336	0.547	0.54
15	-0.828	-0.581	-0.987	1.028
16	0.933	1.524	0.553	0.791
17	-0.792	-1.857	-0.107	1.023
18	1.139	0.292	1.684	0.504
19	0.905	0.148	1.392	0.534
20	-0.515	0.024	-0.861	0.656
21	-0.066	-0.988	0.526	0.635
22	0.603	0.344	0.770	0.588
23	-0.427	-0.976	-0.074	0.769
24	-0.906	-1.783	-0.342	0.805
25	0.173	-0.330	0.496	0.633
26	-0.190	-0.404	-0.053	0.677
27	-1.213	0.322	-2.199	0.801
28	0.198	0.511	-0.003	0.499
29	1.570	0.339	2.361	0.775
30	0.586	-0.162	1.067	0.613
31	-1.358	0.288	-2.416	0.863
32	0.411	-0.896	1.251	0.608
33	-1.367	-1.859	-1.051	0.955
34	-0.339	-0.161	-0.454	1.004
35	0.366	-0.228	0.748	0.724
36	-0.399	-0.895	-0.081	0.705
37	0.841	0.916	0.792	0.561
38	-1.450	-0.251	-2.220	1.042
39	-1.665	-1.051	-2.060	1.236
40	-0.999	-1.144	-0.905	1.054

41	0.186	-0.663	0.732	0.891
42	1.205	0.485	1.667	0.516
43	0.185	0.501	-0.018	0.619
44	0.047	0.169	-0.031	0.632
45	1.103	-0.061	1.851	0.564
46	0.707	-0.717	1.622	0.786
47	-0.975	0.894	-2.177	0.683
48	0.092	1.489	-0.806	0.491
49	-1.578	-0.560	-2.232	1.189
50	0.967	-0.088	1.646	0.632
